# Stress responses, vitagenes and hormesis as critical determinants in aging and longevity: Mitochondria as a “chi”

**DOI:** 10.1186/1742-4933-10-15

**Published:** 2013-04-25

**Authors:** Carolin Cornelius, Rosario Perrotta, Antonio Graziano, Edward J Calabrese, Vittorio Calabrese

**Affiliations:** 1 Department of Chemistry University of Catania, Viale Andrea Doria, 95100 Catania, Italy; 2 Department of Medicine and Surgery, University of Catania, Viale Andrea Doria, Catania, 95100, Italy; 3 Environmental Health Sciences Division, School of Public Health, University of Massachusetts, Amherst, MA, USA

## Abstract

Understanding mechanisms of aging and determinants of life span will help to reduce age-related morbidity and facilitate healthy aging. Average lifespan has increased over the last centuries, as a consequence of medical and environmental factors, but maximal life span remains unchanged. Extension of maximal life span is currently possible in animal models with measures such as genetic manipulations and caloric restriction (CR). CR appears to prolong life by reducing reactive oxygen species (ROS)-mediated oxidative damage. But ROS formation, which is positively implicated in cellular stress response mechanisms, is a highly regulated process controlled by a complex network of intracellular signaling pathways. By sensing the intracellular nutrient and energy status, the functional state of mitochondria, and the concentration of ROS produced in mitochondria, the longevity network regulates life span across species by coordinating information flow along its convergent, divergent and multiply branched signaling pathways, including vitagenes which are genes involved in preserving cellular homeostasis during stressful conditions. Vitagenes encode for heat shock proteins (Hsp) Hsp32, Hsp70, the thioredoxin and the sirtuin protein systems. Dietary antioxidants, have recently been demonstrated to be neuroprotective through the activation of hormetic pathways, including vitagenes. The hormetic dose–response, challenges long-standing beliefs about the nature of the dose–response in a lowdose zone, having the potential to affect significantly the design of pre-clinical studies and clinical trials as well as strategies for optimal patient dosing in the treatment of numerous diseases. Given the broad cytoprotective properties of the heat shock response there is now strong interest in discovering and developing pharmacological agents capable of inducing stress responses. Here we focus on possible signaling mechanisms involved in the activation of vitagenes resulting in enhanced defense against energy and stress resistance homeostasis dysiruption with consequent impact on longevity processes.

## Introduction

Western Medicine is in crisis. Continually increasing resources are being expended to combat the age-related diseases that include diabetes and metabolic syndrome, Alzheimer disease, and cancer. Yet, the causes of these disease remain a mystery, while their incidence and morbidity either remain constant or are increasing. Huge investments in biomedical research in the recent past have resulted in some striking accomplishments, including the sequencing of the human chromosomal single nucleotide polymorphisms (SNPs), and the identification of regional clusters of chromosomal SNPs (the HapMap). However, these accomplishments have failed to reveal the anticipated genetic causes for the common age-related diseases [1-10].

While the anatomical paradigm of medicine and the Mendelian paradigm of genetics have been powerful predictors of medical relationships for the past century, they are failing to direct us toward solutions for the common age-related disease. When a prevailing paradigm fails to make productive predictions, then the hypothesis-based research begins to fail. To resolve this crisis window and to return to productive “normal science”, a new paradigm must be generated which encompasses the failing strengths of the previous paradigm but adds new elements addressing the current problems being confronted. Assuming that this analysis is applicable to biomedical sciences, what could be the missing components of the anatomical and mendelian paradigms necessary for understanding the age-related diseases remains still elusive. Mitochondrial biology and genetics are being recognized as powerful candidate for expanding anatomical and Mendelian paradigms to address the complexities of the age-related diseases, aging and cancer [11-15].

Life involves the interplay between structure and energy. For the eukaryotic cell, this duality was cemented approximately 2 billion years ago (earth is approximately 5 billion years old) by the symbiosis of what seems to have been a glycolitic motile cell, which gave rise to the nucleus-cytosol, and an oxidative proteobacterium, which evolved into the mitochondrion. Initially each organism was free living and contained all the genes for an independent life. However. Over the subsequent 1.2 billion of years, the single cell descendants of the original symbiosis experimented many alternative rearrangements of biomedical interdependence and genomic reorganization. This, ultimately, an arrangement was achieved in which the mitochondrion became specialized in energy production and the nucleus-cytosol polarized his specialization twards structure. This final design prompted impetuously the development of multicellularity and the evolution of higher plants and animals, including humans. This original design powerfully comeback anytime in our cell when tumor promotion occurs, whereby the glycolitic imperatively dictates a kicking off process of mitochondrial energy process marginalitation, in favour of sustained high proliferative potential of tumor progression. In this scenario mitochondria acts as the ancient concept of Asian medicine where “chi” stands for vital force of energy [16-22].

Thus, understanding mechanisms of aging and determinants of life span will help to reduce age-related morbidity and facilitate healthy aging. Average lifespan has increased over the last centuries, as a consequence of medical and environmental factors, but maximal life span remains unchanged [23]. Extension of maximal life span is currently possible in animal models with measures such as genetic manipulations and caloric restriction (CR). CR appears to prolong life by reducing reactive oxygen species (ROS)-mediated oxidative damage. But ROS formation, which is positively implicated in cellular stress response mechanisms, is a highly regulated process controlled by a complex network of intracellular signaling pathways. By sensing the intracellular nutrient and energy status, the functional state of mitochondria, and the concentration of ROS produced in mitochondria, the longevity network regulates life span across species by coordinating information flow along its convergent, divergent and multiply branched signaling pathways, including vitagenes which are genes involved in preserving cellular homeostasis during stressful conditions. Vitagenes encode for heat shock proteins (Hsp) Hsp32, Hsp70, the thioredoxin and the sirtuin protein systems [23]. Dietary antioxidants, have recently been demonstrated to be neuroprotective through the activation of hormetic pathways, including vitagenes. The hormetic dose–response, challenges long-standing beliefs about the nature of the dose–response in a lowdose zone, having the potential to affect significantly the design of pre-clinical studies and clinical trials as well as strategies for optimal patient dosing in the treatment of numerous diseases [23]. Given the broad cytoprotective properties of the heat shock response there is now strong interest in discovering and developing pharmacological agents capable of inducing stress responses. In this review we discuss the most current and up to date understanding of the possible signaling mechanisms by which caloric restriction, as well hormetic caloric restriction-mimetics compunds by activating vitagenes can enhance defensive systems involved in bioenergetic and stress resistance homeostasis with consequent impact on longevity processes [24-30].

### Hormesis and Longevity

**Figure 1:**
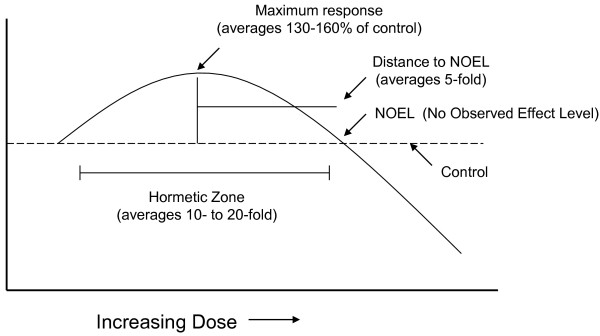
Quantitative features of hormetic dose-response curve.

Hormesis is a dose response phenomenon characterized by a low dose stimulation and a high dose inhibition (Figure 1). It may be graphically represented by either an inverted U-shaped dose response or by a J- or U-shaped dose response. The term hormesis was first presented in the published literature in 1943 by Southam and Ehrlich who reported that low doses of extracts from the Red Cedar tree enhanced the proliferation of fungi with the overall shape of the dose response being biphasic. However, credit for experimentally demonstrating the occurrence of hormesis belongs to Hugo Schulz (1888) [23] who reported biphasic dose responses in yeast following exposure to a large number of chemical disinfectant agents. The work of Schulz encouraged numerous investigators in diverse fields to assess whether such low dose effects may be a general feature of biological systems. In fact, similar types of dose response observations were subsequently reported by numerous researchers assessing chemicals [31] and radiation [32-34] with investigators adopting different names such as the Arndt-Schulz Law, Hueppe’s Rule, and other terms to describe these similar dose response phenomena. Despite the rather substantial historical literature concerning hormetic dose responses, this concept had a difficult time being incorporated into routine hazard assessment and pharmacological investigations, principally because it (i) required more rigorous evaluation in the low dose zone, (ii) failure of investigators to understand its clinical significance (iii) failure to appreciate the quantitative features of the hormetic dose response (iv) failure to understand the limitations of its agricultural and industrial applications, (v) because of the predominant interest in responses at relatively high doses during most of the 20^th^ century as well as (vi) the continuing, yet inappropriate, tendency to associate the concept of hormesis with the medical practice of homeopathy [35]. However, since the late 1970’s there has been a growing interest in hormetic-like biphasic dose responses across the broad spectrum of biomedical sciences. This resurgence of interest resulted from a variety of factors, including the capacity to measure progressively lower doses of drugs and chemicals, the adoption of cell culture methods which has permitted more efficient testing of numerous doses and the need to re-examine the validity of linear at low dose modelling of cancer risks due to their enormous cost implications for environmental regulations, as well the astute observations of numerous independent investigators to generalize their hormetic findings across biological systems [36,37].

The concept of hormesis is a central to the biomedical sciences because of its generalizability [23,34,38]. Hormetic dose responses are independent of biological model, endpoint as well as chemical class and physical agent [23]. In direct head to head comparisons with the threshold and linear dose response models the hormetic dose response model was found in multiple studies with many thousands of dose responses to make far more accurate predicts of low dose responses [38]. The hormetic dose response has also been shown to describe the fundamental features of several dozen receptor systems, affecting a vast array of biological endpoints [34]. The concepts of pre-conditioning and post-conditioning are also manifestations of hormesis. When these two highly significant and general concepts are analyzed within the dose response framework they conform to the features of the hormetic dose response [31]. Of particular importance is that the quantitative features of the hormetic dose response are similar across biological models and endpoints. This remarkable general feature of hormetic dose response suggests that the hormetic dose response may represent the first comprehensively based quantitative estimation of biological plasticity [35].

The failure to recognize and incorporate the concept of hormesis as a central biological concept during the key development period of dose response concept consolidation for pharmacology and toxicology during the middle decades of the 20^th^ century had many implications. For example, the failure to recognize the generality of biphasic dose responses lead to the development of toxicology becoming a high dose testing discipline that also only tested few doses. It became a discipline that developed flawed regulations based on an inadequate understanding of the dose response that have inaccurately estimated the costs and the benefits of such regulations, thereby yielding an inadequate foundation for public policy decisions [23]. Such historical failures lead not only to the development of biologically unsupportable environmental regulations especially for carcinogens but also profoundly slowed the recognition and generality of adaptive responses and how they might be exploited within society. Not only was this failure highly significant in the environmental realm but it also lead to the failure of the biomedical community to adequately test chemotherapeutic agents adequately. For example, many anti-tumor agents that kill tumor cells at high doses may also stimulate the proliferation of these cells at lower doses, displaying the hormetic dose response [38]. Furthermore, since the hormetic dose response has clearly defined quantitative features and is highly generalizable it also provides a quantitative estimate of biological performance. That is, it estimates the extent to which memory drugs can enhance learning. This concept can be readily applied to all other areas of biological performance such as bone strengthening, hair growth, decreases in anxiety, plant growth and productivity and others.

Of particular relevance to the current paper is that the hormesis concept can be used to understand the biological limits within which pharmaceutical efforts are made to enhance the quality of aging and to affect longevity. For example, the hormesis concept argues that the biological performance can only be modestly improved in all biological systems in most situations. The term modestly is quantitatively constrained to be less than two fold, with most maximal increases being within the 30-60% range. Such biological constraints are of vital importance to pharmaceutical companies and regulatory agencies as they develop strategies for product development and evaluation. This also suggests that attempts to extend life would also be constrained within the bounds of plasticity, which are quantitatively estimated by the hormetic dose response. Thus, if the normal bounds of human longevity are seen to be approximately as about 100 years, the hormesis concept predicts that it may be possible to extend the human lifespan by 30–60 years at most.

The data to support these types of biological constrains are extensive. They are based on more than 20,000 dose responses in several hormesis databases. These data sets are very general, including responses across a very broad range of biological models and endpoints to a very large number of chemical agents representing a broad spectrum of chemical classes. The range and the scope of these findings have come a long way in clarifying the limits within which biological systems operate, the overlapping features of biological control redundancies, and the constraints and flexibilities within which cells, organs and organisms operate. All of these features affect the extent to which humans can manipulate biological systems and attempt to gain improvement in function and performance. Thus, the subsequent sections of this paper on cellular stress responses and vitagenes all can and should be seen within an hormetic context. Even the concept of wound healing, which is essential to ensuring survival and enhancing longevity are fundamentally described within an hormetic context.

One of the best examples in rodents for chemical-induced prolongation of life involves the effects of (-)deprenyl. The life prolonging effects of (-)deprenyl has been demonstrated in at least four different animal species [23,34,38]. For example, (-)deprenyl has extended the lifespan in male Logan-Wistar rats, male F-344 rats, dogs, and mice and hamsters [23]. (-)Deprenyl has also been shown to decrease longevity as well in a number of species. When the toxicological findings of these numerous studies was rationalized, what emerged was consistent with the dose response pattern in which there was enhancement of survival and prolongation of life at lower doses but also a decrease in longevity at the higher doses. It was concluded that the low dose enhanced prolongation of lifespan by (-)deprenyl was an example of hormesis. Follow up evaluations of (-)deprenyl on axtioxidant enzymes (i.e., catalase, total SOD, CuZn SOD and Mn SOD) one month before the end of a 27 month longevity study all reflected the hormetic biphasic dose response activities in the striatum and cortex areas of the brain. These enzymatic findings parallel the longevity results, thus a possible causal association between the anti-oxidant cellular activities and longevity was suggested together with possible mechanism to account for the hormetic effects. While more research is needed to clarify the mechanisms for the enhanced longevity at low dose, these findings also revealed that the extensions of longevity were about 30-50% greater than controls, observations fully consistent with the hormetic dose response [23,34,38].

### Cellular stress response, HSF biology and the Vitagene network

**Figure 2:**
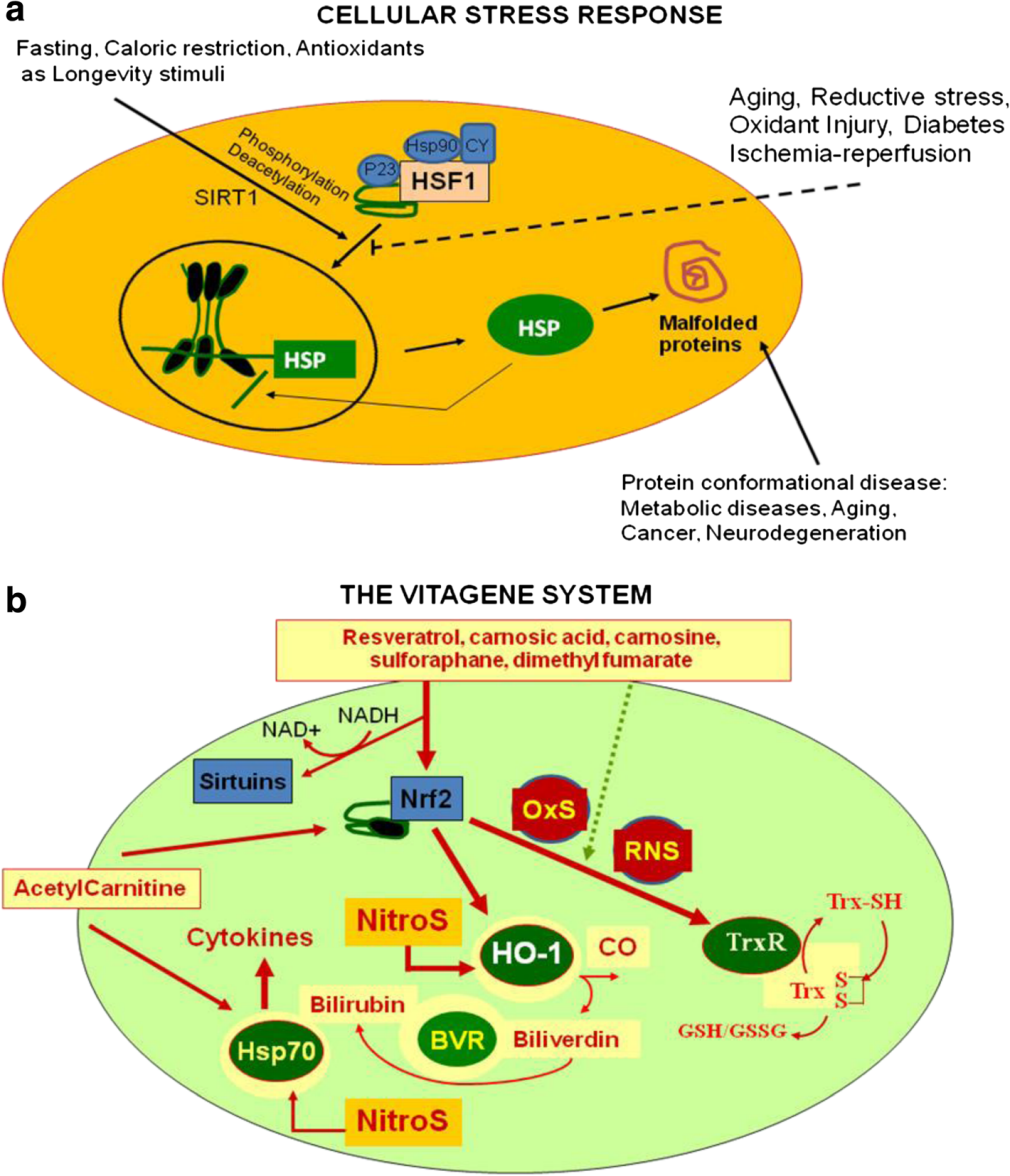
**a,b. Vitagenes and the pathway of cellular stress response. **Cumulating misfolded proteins in response to proteotoxic environmental stress conditions triggers the cellular stress response (Figure 2a). HSPs that are normally bound to HSF1, maintaining it in a repressed state before stress, are titrate away by damaged or misfolded proteins with resulting HSF-1 activation. Multi-step activation of HSF1 involves post-translational modifications, such as hyperphosphorylation and deacetylation, which allow HSF1 to trimerize, translocate into the nucleus, and bind to heat-shock elements (HSEs) in the promoter regions of its target hsp genes. Nutritional antioxidants, including carnosic acid, resveratrol, sulforaphane, dimethyl fumarate, acetyl-L-carnitine or carnosine are able to activate vitagenes, such as heme oxygenase, Hsp70, thioredoxin reductase and sirtuins which represent an integrated system for cellular stress tolerance. Phytochemicals and Acetyl-L-carnitine act through the activation of the vitagene system, with up-regulation of HO-1, Thioredoxin, the GSH and Sirtuin system, results in counteraction of pro-oxidant conditions (Figure 2b). During aging, a gradual decline in potency of the heat shock response occur and this may prevent repair of protein damage, leading to degeneration and cell death.

Cellular stress response is the ability of a cell to counteract stressful conditions (Figure 2a, Figure 2b). This phenomenon, which includes heat shock response (HSR), represents an ancient and highly conserved cytoprotective mechanism [39-47]. Production of heat shock proteins, including protein chaperones, is essential for the folding and repair of damaged proteins, serving thus to promote cell survival conditions that would otherwise result in apoptosis [36,48-51]. The term ‘molecular chaperone’ denotes a large family of ubiquitous proteins that function as part of an ancient defense system in our cells. Chaperones promote cell survival by sequestering damaged proteins and preventing their aggregation. During stressful conditions, such as elevated temperature, they prevent protein aggregation by facilitating the refolding or elimination of misfolded proteins. The stress-induced response to damaged proteins is helped by a sophisticated regulatory system, which shuts down most cellular functions and, in parallel, induces the synthesis of several chaperones and other survival-promoting proteins. Therefore, many of the chaperones are also called stress or ‘heat shock’ proteins in reference to the archetype of cellular stress, heat shock. Besides their role during stress, chaperones have multiple roles under normal conditions, as such they promote the transport of macromolecules (e.g. proteins or RNA) and participate in remodelling events involving larger protein complexes, including signaling, transcription, cell division, migration and differentia. Cellular stress response requires the activation of pro-survival pathways which, under control of protective genes called vitagenes [37] produce molecules (heat shock proteins, glutathione, bilirubin) endowed with anti-oxidant and anti-apoptotic activities. Generally, molecular chaperones help a multitude of signaling molecules to keep their activation-competent state, and regulate various signaling processes ranging from signaling at the plasma membrane to transcription. In addition to these specific regulatory roles, recent studies have revealed that chaperones act as genetic buffers stabilizing the phenotypes of various cells and organisms. Among the cellular pathways conferring protection against oxidative stress, a key role is played by the products of vitagenes [52-54]. These include members of the heat shock protein (Hsp) family, such as heme oxygenase-1 and Hsp72, sirtuins and the thioredoxin/thioredoxin reductase system [55]. Recent studies have shown that the heat shock response contributes to establishing a cytoprotective state in a wide variety of human diseases, including inflammation, cancer, aging and neurodegenerative disorders [56]. Given the broad cytoprotective properties of the heat shock response there is now strong interest in discovering and developing pharmacological agents capable of inducing the heat shock response [57]. Molecular chaperones are known to disrupt aggregates but also to promote active aggregation when the concentration of the aggregating protein is high. Consistent with this notion, although protein aggregation is hazardous under certain circumstances, the creation of apparently less-toxic large aggregates is protective. This hypothesis is the basis of the therapeutic potential of heat shock proteins (HSPs), which prevent protein misfolding and aggregation [58].

Cellular stress response is regulated at the transcriptional, translational and post-translational levels by a family of heat shock transcription factors (HSFs) that are expressed and maintained in an inactive state under non-stress conditions [59]. HSFs, essential for all organisms to survive to acute or chronic stress, are also important for normal development and lifespan-enhancing pathways, and the repertoire of HSF targets has thus expanded well beyond the heat shock genes. Post-translational regulation of HSFs is emerging to integrate the metabolic state of the cell with stress biology, whereby controlling fundamental aspects of the health of the proteome and ageing. At the transcriptional level the heat shock response is mediated by cis-acting sequences called heat shock elements (HSEs) present in multiple copies upstream of the HSP genes. In contrast to a single HSF in invertebrates, multiple HSFs are expressed in plants and vertebrates. Mammalian HSF family consists of four members: HSF1, HSF2, HSF3 and HSF4, which recognize by binding the heat shock element (HSE), composed of inverted repeats of a consensus nGAAn sequence. These distinct HSFs possess overlapping functions, display tissue-specific patterns of expression, undergoing multiple post-translational modifications, and show various interacting protein partners. In addition to thermal stress, the inducible expression of heat shock proteins is also triggered by environmental redox changes or exposure to electrophiles which cause trimerization and DNA binding of HSF1, pointing to the importance of the cysteine redox state for the activation of this transcription factor [59]. Thus, an intermolecular disulfide bond formation between C36 and C103 within HSF1 causes trimerization and DNA binding, whereas an intramolecular disulfide bond formation is inhibitory for the activity of the transcription factor. Hence, pharmacologic modulation of HSF-mediated gene regulation is an emerging area of research which is increasing its potential based on the current knowledge of small-molecule activators and inhibitors of HSFs, so that the impact of HSFs is further extending beyond the heat shock response, which possesses the potential for attracting growing interest [60,61]. HSFs, like other transcription factors, are composed of functional domains which have been well characterized for HSF1. The DNA-binding domain (DBD) belongs to the family of winged helix-turn-helix DBDs. The DBD, the signature domain of HSFs for recognition of target-genes, forms a compact globular structure, except for a flexible wing or loop, located between β-strands 3 and 4, which is responsible for protein-protein interactions between adjacent subunits of the trimerized HSF, conferring high-affinity binding to DNA and mediating interactions with other factors to modulate the transactivating capacity of HSFs. The trimerization of HSFs is mediated by arrays of hydrophobic heptad repeats (HR-A and HR-B) forming coiled coil, characteristic for many Leu zippers [61]. Trimerization of HSF, facilitated by leu zippers is abrogated by another hydrophobic repeat, HR-C located between the regulatory and trans-activation domains (TADs). This latter, by interacting with the HR-A/B domain prevents trimerization maintaining HSF1 in an inactive status. The transactivation domain in all HSFs is positioned at the extreme amino terminus, and is composed of two modules — AD1 and AD2, which are rich in hydrophobic and acidic residues which allow rapid and prolonged response to stress (**61 57**). Although the N-terminal parts of HSF1 mediate trimer formation and acquisition of DNA-binding ability, the C-terminal parts of the protein facilitate transcriptional activation of target genes and also regulate the magnitude of HSF1 activation [59,61]. Although the regulatory domain of HSF has an intrinsic competence to sense heat stress, its inducibility is modulated through posttranslational modifications [23,61]. Pharmacological inhibition of Hsp90 activity is sufficient to convert HSF1 into a DNA-binding trimeric state in cells, thus in nonstressed cells, HSF1 is maintained in an inactive monomeric state by interaction with Hsp90. This interaction is rapidly reduced in cells exposed to proteotoxic insults, where binding of HSF1 to the promoter occurs within seconds and in about one minute is saturated by the intervent of other positive signals such as a ribonucleoprotein complex containing eukaryotic elongation factor 1A (eEF1A) and a constitutive non-coding RNA, heat shock RNA-1 (HSR-1), which has been reported to possess a thermosensing capacity [62]. Consistent with the proposed model, HSR-1 undergoes a conformational change in response to heat stress facilitating, together with eEF1A, HSF1 trimerization. In this activation cycle, HSF1 undergoes extensive post translational modifications, such as phosphorylation, sumoylation and acetylation. Under unstressed conditions HSF1 is a phosphoprotein with at least 12 Ser residues are phosphorylated (S121, S230, S292, S303, S307, S314, S319, S326, S344, S363, S419, and S444), with no detectable amount of threonine or tyrosine phosphorylation. Phosphorylation-dependent sumoylation of HSF1 on K298 requires a priming phosphorylation of S303. Phosphorylation of S303, possibly mediated by MAP kinases (MAPK), facilitates HSF1 sumoylation by the SUMO E2 conjugating enzyme Ubc9, leading to repression of HSF1 trans-activating capacity. SUMO proteins resemble structurally to ubiquitin and are transiently and covalently bound to specific lysine (K) residues of multiple cellular proteins. In many cases, sumoylation occurs on a consensus sequence consisting of the tetrapeptide ΨKxE, in which Ψ is represented by a branched hydrophobic amino acid (usually a leucin, isoleucin, or valine) and K the SUMO acceptor. In HSF1, adjacent consensus sequences for sumoylation ΨKxE and phosphorylation (SP) together form an extended target motif, ΨKxESP (PDSM), mediating phosphorylation-dependent sumoylation, acting not only in the regulation of HSF biology, but also in a wide range of transcriptional regulators, where SUMO proteins are well-established repressors of transcription. While phosphorylation and sumoylation of HSF1 occur rapidly on heat shock, the kinetics of acetylation are delayed and coincide with the attenuation phase of the HSF1 activation cycle. Consistent with this notion, acetylation of HSF1 is regulated by the balance of acetylation by p300–CBP (CREB-binding protein) and deacetylation by the NAD + -dependent sirtuin, SIRT1. Increased expression and activity of SIRT1 enhances and prolongs the DNA-binding activity of HSF1 at the human HSP70.1 promoter, whereas downregulation of SIRT1 enhances the acetylation of HSF1 and the attenuation of DNA-binding without affecting the formation of HSF1 trimers [23]. Thus SIRT1 maintains HSF1 in a state that is competent for DNA binding by counteracting acetylation . In the light of current knowledge, the attenuation phase of the HSF1 cycle is regulated by a dual mechanism: a dependency on the levels of HSPs that feed back directly by weak interactions with HSF1, and a parallel step that involves the SIRT1-dependent control of the DNA-binding activity of HSF1. Because SIRT1 has been implicated in caloric restriction and ageing, the age-dependent loss of SIRT1 and impaired HSF1 activity correlate with an impairment of the heat shock response and proteostasis in senescent cells, connecting the heat shock response to nutrition and ageing [23].

#### Keap1/Nrf2/ARE biology and the Heme oxygenase pathway of Stress tolerance

As mentioned above, a central regulator in the gene expression of heat shock proteins is HSF1, however, in addition to this some of the vitagenes are also upregulated within the “phase 2 response”, known as “the electrophile counterattack response”, a cytoprotective response that protects against various electrophiles and oxidants [20-23]. Examples include heme oxygenase 1, thioredoxin and thioredoxin reductase, all of which can be upregulated by the transcription factor Nrf2 (Nuclear erythroid 2-related factor 2) co-ordinately with a battery of cytoprotective proteins, such as glutathione transferases (GST), UDP-glucuronosyltransferase, NAD(P)H:quinone oxidoreductase 1 (NQO1), epoxide hydrolase, ferritin, γ-glutamylcysteine synthetase, glutathione reductase, aldo-keto reductases, and glutathione conjugate efflux pumps [20-23]. This elaborate network of protective mechanisms allows eukaryotic organisms to counteract the damaging effects of oxidants and electrophiles, major agents involved in the pathogenesis of cancer, atherosclerosis, neurodegeneration, and aging. The gene expression of these cytoprotective proteins is coordinately regulated by a common molecular mechanism that involves the Keap1/Nrf2/ARE pathway. The upstream regulatory regions of these genes contain single or multiple copies of the antioxidant/electrophile response elements (ARE, EpRE) with the consensus sequence 5’-A/CTGAC/GNNNGCA/G-3’). The major transcription factor that binds to the ARE is nuclear factor erythroid 2-related factor 2 (Nrf2), a basic leucine zipper transcription factor. Activation of gene expression requires that Nrf2 binds to the ARE in heterodimeric combinations with members of the small Maf family of transcription factors. Under basal conditions the pathway operates at low levels due to the repressor function of the cytosolic protein Kelch-like ECH-associated protein 1 (Keap1), which binds to the E3 ubiquitin ligase Cullin3-RING box1 (Cul3-Rbx1) and presents Nrf2 for ubiquitination and subsequent proteosomal degradation. Inducers of the Keap1/Nrf2/ARE pathway belong to at least ten distinct chemical classes: (i) oxidizable diphenols, phenylenediamines, and quinones; (ii) Michael acceptors (olefins or acetylenes conjugated to electron-withdrawing groups); (iii) isothiocyanates; (iv) thiocarbamates; (v) trivalent arsenicals; (vi) dithiolethiones; (vii) hydroperoxides; (viii) vicinal dimercaptans; (ix) heavy metals; and (x) polyenes. The only common property among them is their chemical reactivity with sulfhydryl groups by oxido-reduction, alkylation, or disulfide interchange. A large number of in vivo experiments have demonstrated the neuroprotective role of the Keap1/Nrf2/ARE pathway and suggested the potential use of Nrf2 inducers for achieving protection against neurodegenerative diseases. Thus, in comparison to wild-type mice, nrf2-knockout mice are hypersensitive to the mitochondrial complex II inhibitors 3-nitropropionic acid and malonate which induce lesions in the striatum. Nrf2-knockout mice are also more susceptible to seizures, neuronal damage and microglial infiltration in hippocampus induced by kainic acid exposure [20-23]. Nrf2-dependent protection against excess release of dopamine was also recently reported [20-23]. Transplanted Nrf2-overexpressing astrocytes into the mouse striatum prior to challenge with malonate provided dramatic protection against malonate-induced neurotoxicity and remarkably, brain hemispheres receiving Nrf2-producing astrocyte transplants were essentially resistant to malonate toxicity, whereas hemispheres receiving control astrocytes were no different from untransplanted controls. Among the various redox regulated Vitagenes, containing in its promoter region the antioxidant response element (ARE), there has been a growing interest over the last years in the heme oxygenase (HO) system, the family of enzymes that control the initial and rate-limiting steps in heme catabolism [20-23]. The heme oxygenases have been recognized as dynamic sensors of cellular oxidative stress and modulators of redox homeostasis throughout the phylogenetic spectrum. Heme oxygenases are located within the endoplasmic reticulum where they act in association with NADPH cytochrome P450 reductase to oxidize heme to biliverdin, free ferrous iron and carbon monoxide (CO. Biliverdin reductase further catabolizes biliverdin to the bile pigment, bilirubin, a linear tetrapyrrole which has been shown to effectively counteract nitrosative stress due to its ability to interact with NO and RNS [20-23]. Bilirubin is then conjugated with glucuronic acid and excreted [20-23]. Bilirubin has been shown to serve as an endogenous scavenger for both nitric oxide and reactive nitrogen species, which may alter the redox status of the cell and originate nitrosative stress [20-23]. Despite this important antioxidant properties, if produced in excess, as in the case of haemolytic anaemia or sepsis, unconjugated bilirubin becomes neurotoxic through multiple mechanisms involving the disruption of cell membrane structure, the reduction of mitochondrial transmembrane potential and the activation of the apoptotic cascade [20-23]. Mammalian cells express at least two isoforms of HO, HO-1 and HO-2. A third protein, HO-3, determined to be a retrotransposition of the HO-2 gene (pseudogene) has been found unique to rats. Although HO-1 and HO-2 catalyze the same reaction, they play different roles in protecting tissues against injury. A convincing hypothesis suggests that HO-1 induction is one of the earlier cellular response to tissue damage and is responsible for the rapid clearance of the intracellular pro-oxidant heme and its transformation into CO and biliverdin the latter being the precursor of the antioxidant bilirubin. On the contrary, constitutively expressed HO-2 is primarily involved in maintaining cellular heme homeostasis as well as in the sensing of intracellular levels of gaseous compounds including NO and CO. Due to the antioxidant response element (ARE) contained in its promoter region, redox regulation of HO-1 gene is now well defined [20-23]. HO-1, in fact, can be induced by several stimuli associated with oxidative and/or nitrosative stress, such as heme, Aβ, dopamine analogues, H2O2, hyperoxia, UV light, heavy metals, prostaglandins, NO, peroxynitrite, Th1 cytokines, oxidized lipid products and lipopolysaccharide, as well as certain growth factors. The induction of *ho-1* is regulated principally by two upstream enhancers, E1 and E2. Both enhancer regions contain multiple stress (or antioxidant) responsive elements (StRE, also called ARE) that also conform to the sequence of the Maf recognition element (MARE) [63] with a consensus sequence (GCnnnGTA) similar to that of other antioxidant enzymes. Polymorphisms in the lengths of GT repeats [11-40] within the HMOX1 promoter appear to be an important determinant of HO-1 expression and function in humans. Long GT sequences code for relatively unstable (Z-conformational) DNA with attenuated transcriptional activity and diminished baseline and stimulated HO-1 protein expression profiles. Significantly higher HO-1 activity is associated with the short-GT polymorphisms which may protect against atherosclerosis-linked conditions (e.g. coronary artery disease), whereas the malignant behavior of various neoplasms was fairly consistently enhanced by the short-GT form. Due to its strong antioxidant properties and wide distribution within the CNS, HO-1 has been proposed as a key enzyme in the prevention of brain damage [64]. The neuroprotective effects of over-expressed HO-1 can be attributed to: (i) increase in cGMP and bcl-2 levels in neurons; (ii) inactivation of p53, a protein involved in promoting cell death; (iii) increase in antioxidant sources and (iv) increase in the iron sequestering protein, ferritin. Specifically, the interaction between HO-1, p53 and Bcl2 could involve the “heme-regulating motifs” of HO-2, which could modulate gene expression by promoting oxygen radical formation and acting as a “sink” for NO. In the absence of elevated intracellular heme or oxidative stress, the basic region leucine zipper transcriptional regulator BACH1 binds HMOX1 antioxidant response elements and represses transcription. Conversely, increased intracellular heme or sulfhydryl oxidation inactivate BACH1, permitting transcriptional induction of HMOX1. Although it is generally agreed that increased HO-1 expression is a common feature during oxidative stress, recent papers demonstrated that HO-1 can be repressed during oxidative stress conditions. In particular human cells exposed to hypoxia, thermal stress and interferon-γ showed a marked HO-1 repression. The importance of HO-1 repression has been corroborated by the discovery of Bach1/Bach2 as heme-regulated transcription factors for HO-1 gene. In fact, Bach1 is broadly expressed in mice and human tissues and, in human cells, it is induced by the same stimuli which are able to repress HO-1 gene [23]. Current hypothesis suggests that HO-1 repression is useful for the cell because (i) decreases the energy costs necessary for heme degradation, (ii) reduces the accumulation of CO and bilirubin which can become toxic if produced in excess and (iii) increases the intracellular content of heme necessary for the preservation of vital functions such as respiration [55,59]. Particularly interesting is the role played by HO-1 in AD, a neurodegenerative disorder which involves a chronic inflammatory response associated with both oxidative brain injury and Aβ-associated pathology. Significant increases in the levels of HO-1 have been observed in AD brains in association with neurofibrillary tangles and also HO-1 mRNA was found increased in AD neocortex and cerebral vessels; the HO-1 increase also co-localized with senile plaques and glial fibrillary acidic protein-positive astrocytes in AD brains. It is plausible that the dramatic increase in HO-1 in AD may be a direct response to an increase in free heme concentrations, associated with neurodegeneration, and can be considered as an attempt of brain cells to convert the highly toxic heme into the antioxidants CO and bilirubin. The protective role played by HO-1 and its products in AD raised new possibilities regarding the possible use of natural substances, which are able to increase HO-1 levels, as potential drugs for the prevention and treatment of AD. In this light very promising are the polyphenolic compounds contained in some herbs and spices, e.g. curcumin. Curcumin is the active anti-oxidant principle in Curcuma longa, a colouring agent and food additive commonly used in Indian culinary preparations. This polyphenolic substance has the potential to inhibit lipid peroxidation and to effectively intercept and neutralize ROS and RNS. In addition, curcumin has been shown to significantly increase HO-1 in astrocytes and vascular endothelial cells. This latter effect on HO-1 can explain, at least in part, the anti-oxidant properties of curcumin, in particular keeping in mind that HO-1-derived bilirubin has the ability to scavenge both ROS and RNS. Epidemiological studies suggested that curcumin, as one of the most prevalent nutritional and medicinal compounds used by the Indian population, is responsible for the significantly reduced (4.4- fold) prevalence of AD in India compared to United States [23,55,59].

Based on these findings, Lim and colleagues have provided convincing evidence that dietary curcumin given to an AD transgenic APPSw mouse model (Tg2576) for 6 months resulted in a suppression of indices of inflammation and oxidative damage in the brain of these mice [65]. Furthermore, increasing evidence indicates that curcumin inhibits NFkB activation, efficiently preventing cell death [64,66,67]. Whereas the acute induction of this enzyme in neural and other tissues is predominantly cytoprotective in nature, protracted or repeated up-regulation of the Hmox1 gene in astrocytes, oligodendroglia and possibly neurons may perpetuate cellular dysfunction and demise in many chronic degenerative and neuroinflammatory conditions long after provocative stimuli iniziation. Within this context, heme-derived free ferrous iron, CO, and biliverdin/bilirubin are all biologically active substances that can either ameliorate or exacerbate neural injury contingently to the specific disease conditions, such as intensity and duration of HO-1 expression and/or the nature of the resulting redox milieu [68-71]. In ‘stressed’ astroglia, HO-1 hyperactivity promotes mitochondrial sequestration of non-transferrin iron and macroautophagy and may thereby contribute to the pathological iron deposition and bioenergetic failure found in most age-related oxidant neurodegenerative disorders. Glial HO-1 expression may impact also cell survival and neuroplasticity by modulating brain sterol metabolism and proteosomal degradation of neurotoxic protein aggregates [72].

### The mitochondrial theory of aging

Mitochondria are membrane-enclosed organelles found in eukaryotic cells where they generate ATP as a source of chemical energy. ATP synthesis occurs through the respiratory or electron transport chain (ETC.) located at the inner mitochondrial membrane, and consists of five protein complexes (Complexes I–V) [13,17,21,31,35,59].

Besides supplying ATP, mitochondria are involved in many other cell functions including the biosynthesis of heme, cholesterol and phospholipids [13,17,21,31,35,59] and initiation of the apoptotic process [13]. According to the endosymbiosis theory, mitochondria are organelles that evolved from purpurbacteria approximately 1.5 billion years ago. Mitochondria have their own genome [31] and can replicate and transcribe their DNA semi-autonomously. Mitochondrial DNA (mtDNA), like nuclear DNA, is constantly exposed to DNA damaging agents. For many years it was thought, in mtDNA repair, that excessively damaged mtDNA molecules were simply degraded and replaced by newly-generated successors copied from undamaged genomes. However, findings now indicate that mitochondria possess the machinery needed to repair genome damage caused by endogenous or exogenous harmful agents. Harman [35,51] suggested that free radicals are involved in the aging process and that mitochondria-derived ROS may influence cellular aging [35,51]. The treatment of IMR-90 fibroblasts with N-tertbutyl hydroxylamine, (an antioxidant recycled by the mitochondrial electron-transport chain) initially gave support to the theory of mitochondrial involvement in cellular senescence. N-tert-butyl hydroxylamine extends fibroblast replicative capacity and delays changes in mitochondrial function due to age by reducing ROS production, preservation of mitochondrial membrane potential and by increasing the cellular GSH/GSSG ratio [35]. A more recent study has demonstrated that mitochondria derived ROS play an important and direct role in the shortening of telomeres and the onset of senescence [59]. Other findings have proposed that mitochondrial dysfunction leads to mitochondrial biogenesis, thereby increasing the number of cell sites for the production of ROS that causes telomere shortening [13,17,21,31,35,59].

On account of its elevated mutagenic propensity, accumulation of mtDNA during life is thought to be a major cause of age-related disease. The lack of introns and protective histones, limited nucleotide excision and recombination DNA repair mechanisms, location in proximity of the inner mitochondrial membrane with exposure to an enriched free radical milieu are all factors contributing to a mutation rate that is 10-fold higher in the mtDNA than in the nuclear DNA (nDNA) [21,35]. Furthermore, considerable evidence suggests that mtDNA mutations increase as a function of age, reaching the highest levels in brain and muscle. It has been reported that more than twenty different types of deletions accumulate in aging human tissues. The first report on an age-related increase in a mtDNA deletion was found in elderly brain and in Parkinson’s disease [35]. This deletion, called the “common deletion”, was observed between 13-bp sequence repeats beginning at nucleotides 8470 and 13447, removing almost a 5-kb region of mtDNA between ATPase 8 and the ND5 genes. The deletion takes place during replication of the mtDNA, the missing sequence encodes for six essential polypeptides of the respiratory chain and 5 tRNAs, and has been associated with several clinical diseases, such as chronic progressive external ophthalmoplegia and Kearns Sayre syndrome. In a comparison with age-matched controls, an association was found between numerous age-related disorders and higher levels of mtDNA mutations. In the central nervous system (CNS), patients with Parkinson’s disease were found to have a 17 times higher level of the common deletion in the striatum, compared to age-matched controls. There is also evidence of higher levels of this deletion in patients with Alzheimer’s disease, parallelling increased levels in the oxidized nucleotide 8-OH-dG [13]. More than 100 mutations of mtDNA have been associated with human diseas [59]. Phenotypic manifestation of mtDNA mutations is extremely broad ranging from oligosymptomatic patients with isolated deafness, diabetes, ophthalmoplegia, etc., to complex encephalomyopathic disorders that may include dementia, seizures, ataxia, stroke-like episode and so on. There is also a wide range of genotype variants, with rearrangements (deletions, duplications) and point mutations affecting protein coding genes, tRNAs and rRNAs. Although there are some broad genotype/phenotype correlations, considerable overlap also occurs. Further research is needed to fully understand the pathogenetic mechanisms involved in the expression of mtDNA mutations. Recent studies have identified mutations of nuclear genes encoding subunits of the respiratory chain, particularly those of complex I. These principally involve infant onset disease with early death. Moreover recent research has shown that the function of the respiratory chain may be impaired by mutations affecting other mitochondrial proteins or as a secondary phenomenon to other intracellular biochemical derangements. An example is Friedreich ataxia where a mutation of a nuclear encoded protein (frataxin), probably involved in iron homeostasis in mitochondria, results in severe deficiency of the respiratory chain in a pattern indicative of free radical mediated damage [21,35,59].

Mutations of nuclear encoded proteins involved in cytochrome oxidase assembly and maintenance have been identified. As predicted, they are associated with severe deficiency of cytochrome oxidase and Leigh syndrome. Defects of intracellular metabolism, particularly with excess-free radical generation including nitric oxide or peroxynitrite, may cause secondary damage to the respiratory chain. This probably plays an important role in Huntington disease, motor neuron disease (amyotrophic lateral sclerosis) and Wilson disease. It is important to consider however that mutation and ROS induced DNA damage are different, and the diseases mentioned above are mostly dependent on genetics and not a causative factor of oxidative stress. Since a common pathway in the pathogenesis of these disorders appears to be defective oxidative phosphorylation, treatments designed to improve respiratory chain function may bring amelioration in the progression of these disorders [23]. These findings establish that a key feature of the aging process is the relationship between age-associated accumulation of mtDNA mutations and bioenergy dysfunction, at least in tissues predominantly composed of postmitotic cells, such as CNS and skeletal muscle [31].

With regard to mitochondrial bioenergetics, a significant decrease in state 3/state 4 ratio has been seen to occur in brain during aging [21]. Since this ratio relates to the coupling efficiency between electron flux through the electron transport chain and ATP production, an increase in state 4 would reduce mitochondrial complexes and consequently increase free radical species production. A decrease in state 3/state 4 respiration during aging has been associated with a significant decrease in cardiolipin content in brain mitochondria [31]. This loss may play a vital role in age-related reduction in mitochondrial function, and appears to be associated with both quantitative and qualitative region-specific protein changes such as decrease of the inner membrane surface, smaller and fewer cristae, decreased fluidity and increased fragility. Is known that functional changes in brain mitochondria are correlated with modifications in cardiolipin composition. Acetylcarnitine fed to old rats increased cardiolipin to levels found in young rats and restored protein synthesis both in the inner mitochondrial membrane and cellular oxidant/antioxidant balance, suggesting that cellular bioenergetics in aged rats may benefit from administration of this compound [35]. It is interesting to note that caloric restriction, a dietary regimen that increases life-span in rodents, maintains the levels of 18:2 acyl side chains and inhibits cardiolipin composition changes [59]. It has also been shown to retard aging associated changes in oxidative damage, mitochondrial oxidant generation and antioxidant defenses observed during aging [13,17,21,35,59].

Recent finding indicates that calorie restriction and specifically reduced glucose metabolism induce mitochondrial metabolism to extend life span in a number of model organisms including Saccharomyces cerevisiae, Drosophila melanogaster, Caenorhabditis elegans and possibly mice. In contrast with Harman's free radical theory of aging, these effects may be caused by an increase in ROS formation within the mitochondria leading to an adaptive response that culminates in increased stress resistance thought to ultimately cause a long-term reduction in oxidative stress [31]. This type of retrograde response, termed mitochondrial hormesis or mitohormesis, may be involved in the health-promoting effects of physical exercise in humans and, hypothetically, impaired insulin/IGF-1-signaling in model organisms. Abrogation of this mitochondrial ROS signal by antioxidants consistently impairs the lifespan-extending and health-promoting capabilities of glucose restriction and physical exercise, respectively [31]. In short, ROS are essential signaling molecules needed to promote health and longevity. The concept of mitohormesis therefore provides a common mechanistic denominator for the physiological effects of physical exercise, reduced calorie uptake and glucose restriction [23]. Consistent with this notion, calorie restriction slows the progression of age-related diseases, extending the lifespan of many species and, in addition, dietary supplementation with essential and/or branched chain amino acids (BCAAs) has been recently demonstrated to support cardiac and skeletal muscle mitochondrial biogenesis, thereby preventing oxidative damage and enhancing physical endurance in middle-aged mice, with resulting prolonged survival [73].

### Conclusions and future directions

Mitochondria are major cellular sources (and targets) of free radicals, play key roles in the regulation of calcium homeostasis, and are effectors of the intrinsic apoptotic pathway due to the release of signaling molecules that activate and trigger specific caspase cascades. Mitochondrial dysfunction is inherent in a variety of human disorders from the classical mitochondrial diseases arising from mitochondrial DNA mutations (encephalomyopathies) to those involving mitochondrial signaling pathways to the rest of the cell, modulated by organellar dynamics and culminating in programmed cell death. The role of mitochondria in normal aging has been the focus of extensive research in the last decades and being complementary to and maturing from the free radical theory of aging, to the oxidative stress theory of aging, to the mitochondrial oxidative stress theory of aging, and today addressing the tight co-regulation of mitochondrial energy and redox signaling. Consistent with this notion, the emerging role of the carnitine system in counteracting metabolic disturbances present in neurological diseases has become apparent [12,13]. While carnitine’s most widely known function is its involvement in b-oxidation of fatty acids, it may also have other roles in metabolism. Functions of carnitine and acylcarnitines in fatty acid metabolism, ketosis and buffering of the concentration ratio of acyl-CoA to free CoA, are significant in brain metabolism, particularly under conditions associated with neurodegenerative damage. In this scenario, it appears conceivably the function of the carnitine system as a prototypical vitagene operating at the functional interface of energy distribution between ancestral mechanisms of cell proliferation and differentiation and homeostatic mitochondrial dependent processes of cell survival which require energy for cellular stress response and redox homeostasis [23]. Very importantly, new envisioned role exploited for mitochondrial targeted compounds, such as nutritional antioxidants, carnitines or carnosine which, by intersecting convergent mechanisms that rely on cellular energy distribution and availability, such as cellular stress response pathways, DNA repair and molecular fidelity mechanisms, as well as maintenance of optimum antioxidant potential appear to be a promising novel therapeutic approach for those pathophysiological conditions, such as neurodegeneration or cancer, where hormetic stimulation of the vitagene pathway is strongly warranted. Healthy ageing involves the interaction between genes, the environment, and lifestyle factors, particularly diet. Besides evaluating specific gene-environment interactions in relation to exceptional longevity, it is important to focus attention on modifiable lifestyle factors such as diet and nutrition to achieve extension of health span. Thus, a better understanding of nutrient-sensing pathways that have been proven to have a pivotal role in the regulation of life span [74], a better characterization of metobolomics and dietary patterns in centenarians will help to better understand human longevity and assist in the design of strategies to extend the duration of optimal human health.

## Competing interests

The authors declare that they have no competing interests.

## Authors’ contributions

EC contributed to the Hormesis and longevity part; CC, RP, AG and VC contributed to the remaining part. All authors read and approved the final manuscript.

## References

[B1] Calabrese V Butterfield DA Scapagnini G Stella AM Maines MD Redox regulation of heat shock protein expression by signaling involving nitric oxide and carbon monoxide: relevance to brain aging, neurodegenerative disorders, and longevity Antioxid Redox Signal 2006 8 444 477 10.1089/ars.2006.8.444 16677090

[B2] Lodi R Tonon C Calabrese V Schapira AH Friedreich's Ataxia: From Disease Mechanisms to Therapeutic Interventions Antioxid Redox Signal 2006 8 438 443 10.1089/ars.2006.8.438 16677089

[B3] Calabrese V Colombrita C Sultana R Scapagnini G Calvani M Butterfield DA Stella AM Redox modulation of heat shock protein expression by acetylcarnitine in aging brain: relationship to antioxidant status and mitochondrial function Antioxid Redox Signal 2006 8 404 416 10.1089/ars.2006.8.404 16677087

[B4] Scapagnini G Colombrita C Amadio M D'Agata V Arcelli E Sapienza M Quattrone A Calabrese V Curcumin activates defensive genes and protects neurons against oxidative stress Antioxid Redox Signal 2006 8 395 403 10.1089/ars.2006.8.395 16677086

[B5] Calabrese V Maines MD Antiaging medicine: antioxidants and aging Antioxid Redox Signal 2006 8 362 364 10.1089/ars.2006.8.362 16677082

[B6] Poon HF Calabrese V Calvani M Butterfield DA Proteomics analyses of specific protein oxidation and protein expression in aged rat brain and its modulation by L-acetylcarnitine: insights into the mechanisms of action of this proposed therapeutic agent for CNS disorders associated with oxidative stress Antioxid Redox Signal 2006 8 381 394 10.1089/ars.2006.8.381 16677085

[B7] Abdul HM Calabrese V Calvani M Butterfield DA Acetyl-L-carnitine-induced up-regulation of heat shock proteins protects cortical neurons against amyloid-beta peptide 1-42-mediated oxidative stress and neurotoxicity: Implications for Alzheimer's disease J Neurosci Res 2006 84 398 408 10.1002/jnr.20877 16634066

[B8] Perluigi M Joshi G Sultana R Calabrese V De Marco C Coccia R Cini C Butterfield DA In vivo protective effects of ferulic acid ethyl ester against amyloid-beta peptide 1-42-induced oxidative stress J Neurosci Res 2006 84 418 426 10.1002/jnr.20879 16634068

[B9] Mancuso C Perluigi M Cini C De Marco C Giuffrida Stella AM Calabrese V Heme oxygenase and cyclooxygenase in the central nervous system: A functional interplay J Neurosci Res 2006 84 1385 1391 10.1002/jnr.21049 16998916

[B10] Calabrese V Sultana R Scapagnini G Guagliano E Sapienza M Bella R Kanski J Pennisi G Mancuso C Stella AM Butterfield DA Nitrosative stress, cellular stress response, and thiol homeostasis in patients with Alzheimer's disease Antioxid Redox Signal 2006 8 1975 1986 10.1089/ars.2006.8.1975 17034343

[B11] Mancuso C Pani G Calabrese V Bilirubin: an endogenous scavenger of nitric oxide and reactive nitrogen species Redox Rep 2006 11 207 213 10.1179/135100006X154978 17132269

[B12] Mancuso C Scapagnini G Curro D Giuffrida Stella AM De Marco C Butterfield DA Calabrese V Mitochondrial dysfunction, free radical generation and cellular stress response in neurodegenerative disorders Front Biosci 2007 12 1107 1123 10.2741/2130 17127365

[B13] Calabrese V Guagliano E Sapienza M Panebianco M Calafato S Puleo E Pennisi G Mancuso C Butterfield AD Giuffrida Stella AM Redox regulation of cellular stress response in aging and neurodegenerative disorders: role of vitagenes Neurochem Research 2007 32 757 773 10.1007/s11064-006-9203-y 17191135

[B14] Calabrese V Mancuso C Ravagna A Perluigi M Cini C De Marco C Butterfield DA Giuffrida Stella AM In vivo induction of heat shock proteins in the substantia nigra following L-DOPA administration is associated with increased activity of mitochondrial complex I and nitrosative stress in rats: regulation by glutathione redox state J Neurochem 2007 101 709 717 10.1111/j.1471-4159.2006.04367.x 17241115

[B15] Piroddi M Depunzio I Calabrese V Mancuso C Aisa CM Binaglia L Minelli A Butterfield AD Galli F Oxidatively-modified and glycated proteins as candidate pro-inflammatory toxins in uremia and dialysis patients Amino Acids 2007 32 573 592 10.1007/s00726-006-0433-8 17356806

[B16] Calabrese V Highlight Commentary on “Redox proteomics analysis of oxidatively 3 modified proteins in G93A–SOD1 transgenic mice—A model of 4 familial amyotrophic lateral sclerosis” Free Radical Biol Med 2007 43 160 162 10.1016/j.freeradbiomed.2007.04.012 17603925

[B17] Calabrese V Mancuso C Calvani M Rizzarelli E Butterfield DA Giuffrida Stella AM Nitric Oxide in the CNS: Neuroprotection versus Neurotoxicity Nat Neurosci 2007 8 766 775 10.1038/nrn2214 17882254

[B18] Calabrese V Mancuso C Sapienza M Puleo E Calafato S Cornelius C Finocchiaro M Mangiameli A Di Mauro M Stella AM Castellino P Oxidative stress and cellular stress response in diabetic nephropathy Cell Stress Chaperones 2007 12 299 306 10.1379/CSC-270.1 18229449 PMC2134792

[B19] Athanasiou A Clarke AB Turner AE Kumaran NM Vakilpour S Smith PA Bagiokou D Bradshaw TD Westwell AD Fang L Lobo DN Constantinescu CS Calabrese V Loesch A Alexander SP Clothier RH Kendall DA Bates TE Cannabinoid receptor agonists are mitochondrial inhibitors: a unified hypothesis of how cannabinoids modulate mitochondrial function and induce cell death Biochem Biophys Res Commun 2007 364 131 137 10.1016/j.bbrc.2007.09.107 17931597

[B20] Mancuso C Bates TE Butterfield DA Calafato S Cornelius C De Lorenzo A Dinkova Kostova AT Calabrese V Natural antioxidants in Alzheimer's disease Expert Opin Investig Drugs 2007 16 1921 1931 10.1517/13543784.16.12.1921 18042001

[B21] Calabrese V Cornelius C Mancuso C Pennisi G Calafato S Bellia F Bates TE Giuffrida Stella AM Schapira T Dinkova Kostova AT Rizzarelli E Cellular Stress Response: A Novel Target for Chemoprevention and Nutritional Neuroprotection in Aging, Neurodegenerative Disorders and Longevity Neurochem Res 2008 33 2444 2471 10.1007/s11064-008-9775-9 18629638

[B22] Mancuso C Capone C Ranieri SC Fusco S Calabrese V Eboli ML Preziosi P Galeotti T Pani G Bilirubin as an endogenous modulator of neurotrophin redox signaling J Neurosci Res 2008 86 1212 1230 10.1002/jnr.21665 18338802

[B23] Calabrese V Cornelius C Cuzzocrea S Iavicoli I Rizzarelli E Calabrese EJ Hormesis, cellular stress response and vitagenes as critical determinants in aging and longevity Mol Aspects Med 2011 32 279 304 10.1016/j.mam.2011.10.007 22020114

[B24] Calabrese V Signorile A Cornelius C Mancuso C Scapagnini G Ventimiglia B Ragusa N Dinkova-Kostova A Practical approaches to investigate redox regulation of heat shock protein expression and intracellular glutathione redox state Methods Enzymol 2008 441 83 110 18554531 10.1016/S0076-6879(08)01206-8

[B25] Calabrese V Bates TE Mancuso C Cornelius C Ventimiglia B Cambria MT Di Renzo L De Lorenzo A Dinkova-Kostova AT Curcumin and the cellular stress response in free radical-related diseases Mol Nutr Food Res 2008 52 1062 1073 10.1002/mnfr.200700316 18792015

[B26] Calabrese V Calafato S Puleo E Cornelius C Sapienza M Morganti P Mancuso C Redox regulation of cellular stress response by ferulic acid ethyl ester in human dermal fibroblasts: role of vitagenes Clin Dermatol 2008 26 358 363 10.1016/j.clindermatol.2008.01.005 18691515

[B27] Di Renzo L Bertoli A Bigioni M Del Gobbo V Premrov MG Calabrese V Di Daniele N De Lorenzo A Body composition and -174G/C interleukin-6 promoter gene polymorphism: association with progression of insulin resistance in normal weight obese syndrome Curr Pharm Des 2008 14 2699 2706 10.2174/138161208786264061 18991689

[B28] Calabrese V Ientile R Cornelius C Scalia M Cambria MT Ventimiglia B Pennini G Mancuso C Butterfield DA Surh YJ, Dong Z, Cadenas E, Packer L Nutritional redox homeostasis and cellular stress response: Differential role of homocysteine and acetylcarnitine Dietary modulation of cell sygnaling pathways 2008 New York, N.Y. (USA): CRC Press

[B29] Calabrese V Calafato S Cornelius C Mancuso C Dinkova-Kostova A Eleuteri AM Heme oxygenase: A master vitagene involved in cellular stress response Enzymes and the Cellular Fight Against Oxidation 2008 Kerala, India: Research Signpost 37/661 (2), Fort P.O., Trivandrum-695 023 ISBN: 978-81-308-0239-8

[B30] Calabrese V Mancuso C Cornelius C Calafato M Ventimiglia B Butterfield DA Dinkova-Kostova AT Rizzarelli E Alvarez S, Evelson P Reactive nitrogen species and cellular stress tolerance in aging and neurodegeneration: Role of vitagenes Free Radical Pathophysiology 2008 Kerala, India: Transworld Research Network 345 367 37/661 (2). ISBN ISBN 978-81-7895-311-3

[B31] Calabrese V Cornelius C Dinkova-Kostova AT Calabrese EJ Mattson MP Cellular stress responses, the hormesis paradigm and vitagenes: novel targets for therapeutic intervention in neurodegenerative disorders Antioxid Redox Signal 2010 13 1763 1811 10.1089/ars.2009.3074 20446769 PMC2966482

[B32] Calabrese EJ Calabrese V Low dose radiation therapy (LD-RT) is effective in the treatment of arthritis: Animal model findings Int J Radiat Biol 2012 [Epub ahead of print] 10.3109/09553002.2013.752595 23176184

[B33] Calabrese EJ Calabrese V Reduction of arthritic symptoms by low dose radiation therapy (LD-RT) is associated with an anti-inflammatory phenotype Int J Radiat Biol 2012 [Epub ahead of print] 10.3109/09553002.2013.752594 23176159

[B34] Calabrese E Iavicoli I Calabrese V Hormesis: Its impact on medicine and health Hum Exp Toxicol 2012 [Epub ahead of print] 10.1177/0960327112455069 23060412

[B35] Calabrese V Cornelius C Giuffrida AM Calabrese EJ Cellular stress responses, mitostress and carnitine insufficiencies as critical determinants in aging and neurodegenerative disorders: role of hormesis and vitagenes Neurochem Res 2010 35 1880 1915 10.1007/s11064-010-0307-z 21080068

[B36] Calabrese EJ Mattson MP Calabrese V Dose response biology: the case of resveratrol Hum Exp Toxicol 2010 29 1034 1037 10.1177/0960327110383641 21115567

[B37] Calabrese EJ Mattson MP Calabrese V Resveratrol commonly displays hormesis:occurrence and biomedical significance Hum Exp Toxicol 2010 29 980 1015 10.1177/0960327110383625 21115559

[B38] Calabrese EJ Iavicoli I Calabrese V Hormesis: why it is important to biogerontologists Biogerontology 2012 13 215 235 10.1007/s10522-012-9374-7 22270337

[B39] Calabrese V Butterfield DA Stella AM Lajtha A, Regino P-PJ, Rossner S Aging and oxidative stress response in the CNS Development and Aging Changes in the Nervous System. Handbook of Neurochemistry and Molecular Neurobiology 2008 3 128 234 ISBN: 978-0-387-32670-2

[B40] Calabrese V Cornelius C Mancuso C Ientile R Giuffrida Stella AM Butterfield DA Uppu RM, Murthy SN, Pryor WA, Parinandi NL Redox Homeostasis and Cellular Stress Response in Aging and Neurodegeneration Free Radical and Antioxidant Protocols (2nd Edition) 2008 LA, USA: Humana Press

[B41] Calabrese V Perluigi M Cornelius C Coccia R Di Domenico F Mancuso C Pennisi G Dinkova-Kostova AT Fraga CG Phenolics in aging and neurodegenerative disorders Phenolic Compounds of Plant Origin and Health: The Biochemistry behind their Nutritional and Pharmacological Value" 2009 NY. USA: Wiley & Sons

[B42] Calabrese V Cornelius C Dinkova-Kostova AT Calabrese EJ Vitagenes, cellular stress response and acetylcarnitine: relevance to hormesis Biofactors 2009 35 146 160 10.1002/biof.22 19449442

[B43] Calabrese V Cornelius C Mancuso C Barone E Calafato S Bates T Rizzarelli E Kostova AT Vitagenes, dietary antioxidants and neuroprotection in neurodegenerative diseases Front Biosci 2009 14 376 397 19273073 10.2741/3250

[B44] Bellia F Calabrese V Guarino F Cavallaro M Cornelius C De Pinto V Rizzarelli E Carnosinase Levels in Aging Brain: Redox State Induction and Cellular Stress Response Antioxid Redox Signal 2009 11 2759 2775 10.1089/ars.2009.2738 19583493

[B45] Calabrese V Cornelius C Rizzarelli E Owen JB Dinkova-Kostova AT Butterfield DA Nitric oxide in cell survival: a Janus molecule Antioxid Redox Signal 2009 11 2717 2739 10.1089/ars.2009.2721 19558211

[B46] Calabrese V Cornelius C Trovato A Cambria MT Lo Cascio MS Di Rienzo L Condorelli D De Lorenzo A Calabrese EJ The hormetic role of dietary antioxidants in free radical-related diseases Curr Pharm Des 2010 16 8778 8883

[B47] Calabrese V Cornelius C Mancuso C Lentile R Stella AM Butterfield DA Redox homeostasis and cellular stress response in aging and neurodegeneration Methods Mol Biol 2010 610 285 308 10.1007/978-1-60327-029-8_17 20013185

[B48] De Lorenzo A Noce A Bigioni M Calabrese V Della Rocca DG Di Daniele N Tozzo C Di Renzo L The effects of Italian Mediterranean organic diet (IMOD) on health status Curr Pharm Des 2010 16 814 824 10.2174/138161210790883561 20388092

[B49] Di Domenico F Perluigi M Butterfield DA Cornelius C Calabrese V Oxidative Damage in Rat Brain During Aging: Interplay Between Energy and Metabolic Key Target Proteins Neurochem Res 2010 35 2184 2192 10.1007/s11064-010-0295-z 20963486

[B50] Perluigi M Di Domenico F Giorgi A Schininà ME Coccia R Cini C Bellia F Cambria MT Cornelius C Butterfield DA Calabrese V Redox proteomics in aging rat brain: Involvement of mitochondrial reduced glutathione status and mitochondrial protein oxidation in the aging process J Neurosci Res 2010 88 3498 3507 10.1002/jnr.22500 20936692

[B51] Calabrese V Cornelius C Maiolino L Luca M Chiaramonte R Toscano MA Serra A Oxidative stress, redox homeostasis and cellular stress response in ménière's disease: role of vitagenes Neurochem Res 2010 35 2208 2217 10.1007/s11064-010-0304-2 21042850

[B52] Di Paola R Impellizzeri D Trovato SA Mazzon E Bellia F Cavallaro M Cornelius C Vecchio G Calabrese V Rizzarelli E Cuzzocrea S Administration of carnosine in the treatment of acute spinal cord injury Biochem Pharm 2011 82 1478 1489 10.1016/j.bcp.2011.07.074 21787762

[B53] Pennisi G Cornelius C Cavallaro MM Trovato SA Cambria MT Pennisi M Bella R Milone P Ventimiglia B Migliore MR Di Renzo L De Lorenzo A Calabrese V Redox regulation of cellular stress response in multiple sclerosis Biochem Pharm 2011 82 1490 1499 10.1016/j.bcp.2011.07.092 21824468

[B54] Scapagnini G Caruso C Calabrese V Therapeutic Potential of Dietary Polyphenols against Brain Ageing and Neurodegenerative Disorders Adv Exp Med Biol 2011 698 27 35 21520701 10.1007/978-1-4419-7347-4_3

[B55] Bellia F Vecchio G Cuzzocrea S Calabrese V Rizzarelli E Neuroprotection in oxidative driven diseases by carnosine Mol Aspects Med 2011 32 258 266 10.1016/j.mam.2011.10.009 22020110

[B56] Siciliano R Barone E Calabrese V Rispoli V Butterfield DA Mancuso C Experimental Research On Nitric Oxide And The Therapy Of Alzheimer Disease: A Challenging Bridge CNS Neurol Disord Drug Targets 2011 10 766 776 10.2174/187152711798072356 21999733

[B57] Zhang Y Ahn YH Benjamin IJ Honda T Hicks RJ Calabrese V Cole PA Dinkova-Kostova AT HSF1-dependent upregulation of Hsp70 by sulfhydryl-reactive inducers of the KEAP1/NRF2/ARE pathway Chem Biol 2011 18 1355 1361 10.1016/j.chembiol.2011.09.008 22118669 PMC3302153

[B58] Westerheide SD Raynes R Powell C Xue B Uversky VN HSF transcription factor family, heat shock response, and protein intrinsic disorder Curr Protein Pept Sci 2012 13 86 103 10.2174/138920312799277956 22044151

[B59] Calabrese V Cornelius C Dinkova-Kostova AT Iavicoli I Di Paola R Koverech A Cuzzocrea S Rizzarelli E Calabrese EJ Cellular stress responses, hormetic phytochemicals and vitagenes in aging and longevity Biochim Biophys Acta 2012 1822 753 783 10.1016/j.bbadis.2011.11.002 22108204

[B60] Akerfelt M Vihervaara A Laiho A Conter A Christians ES Sistonen L Henriksson E Heat shock transcription factor 1 localizes to sex chromatin during meiotic repression J Biol Chem 2012 285 34469 34476 20802198 10.1074/jbc.M110.157552 PMC2966061

[B61] Fujimoto M Hayashida N Katoh T Oshima K Shinkawa T Prakasam R Tan K Inouye S Takii R Nakai A A novel mouse HSF3 has the potential to activate nonclassical heat-shock genes during heat shock Mol Biol Cell 2010 21 106 116 10.1091/mbc.E09-07-0639 19864465 PMC2801703

[B62] Alam J Cook JL How many transcription factors does it take to turn on the heme oxygenase-1 gene? Am J Respir Cell Mol Biol 2007 36 2 166 74 10.1165/rcmb.2006-0340TR 16990612

[B63] Song W Zukor H Lin SH Hascalovici J Liberman A Tavitian A Mui J Vali H Tong XK Bhardwaj SK Srivastava LK Hamel E Schipper HM Schizophrenia-like features in transgenic mice overexpressing human HO-1 in the astrocytic compartment J Neurosci 2012 32 10841 10853 10.1523/JNEUROSCI.6469-11.2012 22875919 PMC6621004

[B64] Calabrese V Cornelius C Leso V Trovato-Salinaro A Ventimiglia B Cavallaro M Scuto M Rizza S Zanoli L Neri S Castellino P Oxidative stress, glutathione status, sirtuin and cellular stress response in type 2 diabetes Biochim Biophys Acta 2012 1822 729 736 10.1016/j.bbadis.2011.12.003 22186191

[B65] Begum AN Jones MR Lim GP Morihara T Kim P Heath DD Curcumin structure-function, bioavailability, and efficacy in models of neuroinflammation and Alzheimer's disease J Pharmacol Exp Ther 2008 326 1 196 208 10.1124/jpet.108.137455 18417733 PMC2527621

[B66] Gupta SC Kim JH Kannappan R Reuter S Dougherty PM Aggarwal BB Role of nuclear factor kappaB-mediated inflammatory pathways in cancer-related symptoms and their regulation by nutritional agents Exp Biol Med (Maywood) 236 658 671 21565893 10.1258/ebm.2011.011028 PMC3141285

[B67] Di Renzo L Bianchi A Saraceno R Calabrese V Cornelius C Iacopino L Chimenti S De Lorenzo A 174G/C IL-6 gene promoter polymorphism predicts therapeutic response to TNF-α blockers Pharmacogenet Genomics 2012 22 134 142 10.1097/FPC.0b013e32834e5e7b 22158445

[B68] Barone E Di Domenico F Sultana R Coccia R Mancuso C Perluigi M Butterfield DA Heme oxygenase-1 posttranslational modifications in the brain of subjects with Alzheimer disease and mild cognitive impairment Free Radic Biol Med 2012 52 2292 2301 10.1016/j.freeradbiomed.2012.03.020 22549002 PMC3377854

[B69] Barone E Mancuso C Di Domenico F Sultana R Murphy MP Head E Butterfield DA Biliverdin reductase-A: a novel drug target for atorvastatin in a dog pre-clinical model of Alzheimer disease J Neurochem 2012 120 135 146 10.1111/j.1471-4159.2011.07538.x 22004509

[B70] Butterfield DA Barone E Di Domenico F Cenini G Sultana R Murphy MP Mancuso C Head E Atorvastatin treatment in a dog preclinical model of Alzheimer's disease leads to up-regulation of haem oxygenase-1 and is associated with reduced oxidative stress in brain Int J Neuropsychopharmacol 2012 15 981 987 10.1017/S1461145711001118 21767440

[B71] Butterfield DA Barone E Mancuso C Cholesterol-independent neuroprotective and neurotoxic activities of statins: perspectives for statin use in Alzheimer disease and other age-related neurodegenerative disorders Pharmacol Res 2011 64 180 186 10.1016/j.phrs.2011.04.007 21536132 PMC3130102

[B72] Schipper HM Heme oxygenase-1 in Alzheimer disease: a tribute to Moussa Youdim J Neural Transm 2011 118 381 387 10.1007/s00702-010-0436-1 20563825

[B73] Valerio A D'Antona G Nisoli E Branched-chain amino acids, mitochondrial biogenesis, and healthspan: an evolutionary perspective Aging (Albany NY) 2011 3 464 478 21566257 10.18632/aging.100322 PMC3156598

[B74] Davinelli S Willcox DC Scapagnini G Extending healthy ageing: nutrient sensitive pathway and centenarian population Immun Ageing 2012 9 9 10.1186/1742-4933-9-9 22524452 PMC3379947

